# Concurrent occurrence of renal cell carcinoma with rhabdoid features in a married couple: a case report

**DOI:** 10.1186/s13104-014-0957-z

**Published:** 2015-01-15

**Authors:** Ryuji Matsumoto, Nobuo Shinohara, Kanako C-Hatanaka, Naoto Kuroda, Kunihiko Tsuchiya, Satoru Maruyama, Takashige Abe, Katsuya Nonomura

**Affiliations:** Department of Renal and Genitourinary Surgery, Hokkaido University Graduate School of Medicine, North-15, West-7, Sapporo, Japan; Department of Pathology, Hokkaido University Hospital, North-14, West-5, Sapporo, Japan; Department of Diagnostic Pathology, Kochi Red Cross Hospital, Kochi, Japan

**Keywords:** Renal cell carcinoma, Rhabdoid features, Married couple, Asbestos

## Abstract

**Background:**

Renal cell carcinoma (RCC) with rhabdoid features is a rare histology and exhibits clinically aggressive behavior. We report a case of a married couple in whom RCC with rhabdoid features concurrently occurred. The rarity of this event suggests that environmental factors may contribute to the etiology of RCC with rhabdoid features.

**Case presentation:**

A 76-year-old Japanese woman was diagnosed with a hypervascular mass in the right kidney and tumor thrombus extending into the right atrium by enhanced computed tomography (CT). She underwent radical nephrectomy and tumor thrombectomy following systemic therapy with the tyrosine kinase inhibitor sunitinib. The histological evaluation denoted clear cell RCC with rhabdoid features. The patient died of cancer 12 months postoperatively. A 76-year-old man, her husband, presented with gross hematuria 2 weeks after his wife had undergone surgery. He had a long history of asbestos exposure. An abdominal CT scan revealed a hypervascular mass in the right kidney and tumor thrombus extending into the inferior vena cava. He also underwent radical nephrectomy and tumor thrombectomy. The histological evaluation also showed clear cell RCC with rhabdoid features. Bone metastasis occurred 12 months postoperatively, but he died of an unrelated cause 18 months after surgery.

**Conclusion:**

Concurrent occurrence of RCC with rhabdoid features may not to be coincidental. Although further studies are warranted, asbestos exposure may contribute to the etiology of clear cell RCC with rhabdoid features.

## Background

In large consecutive series of patients with malignant renal tumors, approximately 3–5% of RCCs showed rhabdoid features [1-4]. Generally, RCCs with rhabdoid features are highly aggressive malignant tumors and are associated with a poor prognosis. Compared with non-rhabdoid RCCs, these tumors are more likely to present at higher grades, twice as likely to undergo extrarenal invasion, and more likely to metastasize [1]. Microscopically, rhabdoid cells are usually associated with a clear cell RCC component and sometimes linked to sarcomatoid changes [1,3,4]. Rhabdoid cells have large, eccentric nuclei and abundant cytoplasm containing eosinophilic inclusions that are strongly positive for vimentin. In this article, we present very rare cases of a married couple in whom clear cell RCC with rhabdoid features and sarcomatoid change concurrently occurred. Coincidental concurrent occurrence is unlikely, suggesting that environment may be an etiologic factor for such a tumor.

## Case presentation

### Case 1

**Figure 1:**
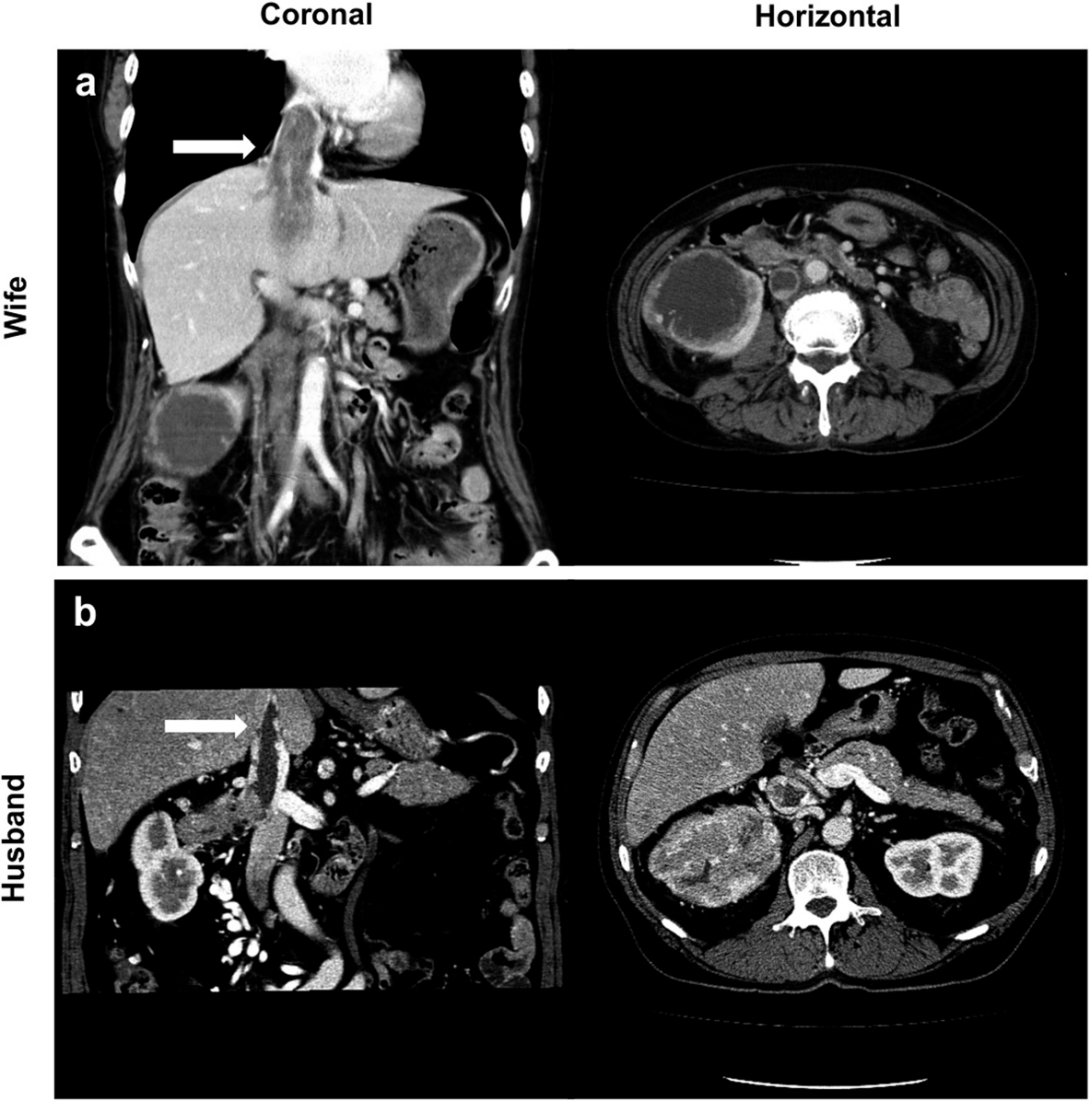
**Right renal tumor and tumor thrombus in a married couple. (a)** Case 1: Abdominal CT revealed that the tumor thrombus extended into the right atrium. **(b)** Case 2 (the husband of Case 1): The tumor thrombus extended into the intrahepatic inferior vena cava.

A 75-year-old womanwas referred to our hospital in May 2009 because enhanced computed tomography (CT) had confirmed the presence of a 70 mm mass on the upper pole of her right kidney and tumor thrombus extending into the right atrium (Figure 1a). Based on a chest CT scan, the patient was initially suspected of having lung carcinoma. Because additional diagnostics were needed to exclude lung carcinoma, systemic therapy was initiated with the tyrosine kinase inhibitor sunitinib. After 4 cycles of sunitinib therapy, tumor regression was observed and the lung tumor had not changed. In December 2009, she underwent right radical nephrectomy and tumor thrombectomy.

**Figure 2:**
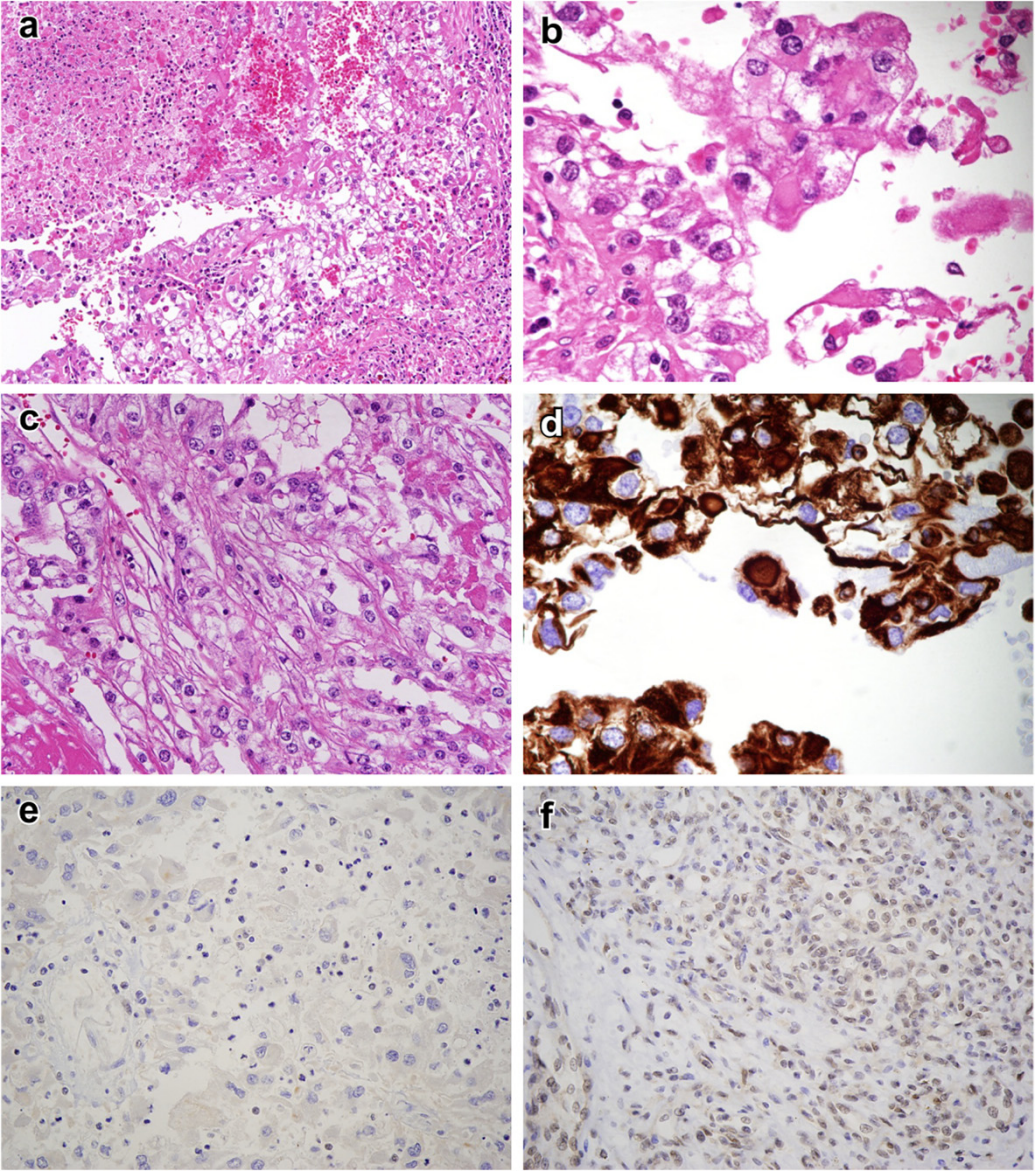
**Pathological findings in Case 1. (a)** Histological section displaying typical clear cell RCC and an area of necrosis, **(b)** neoplastic cells with rhabdoid features, **(c)** spindle neoplastic cells, and **(d)** strong cytoplasmic positivity for vimentin. **(e)** BAP1 immunohistochemistry showed loss of nuclear staining in tumor cells and **(f)** positive control for BAP1 in clear cell RCC.

Postoperatively, the patient developed acute renal failure secondary to circulatory insufficiency, and she received hemodialysis for about a month. The pathological diagnosis of the tumor was clear cell carcinoma with rhabdoid features and sarcomatoid change (assessed in the post-sunitinib therapeutic state; Figure 2a, b, and c). Immunohistochemically, the intracytoplasmic globular structures of rhabdoid cells were positive for vimentin (Figure 2d), and the tumor cell nuclei were mostly negative for BAP1 (Figure 2e and f: positive control of BAP1 in clear cell RCC). In March 2010, pulmonary and abdominal CT scans revealed pulmonary and liver metastasis; therefore, systemic therapy with the tyrosine kinase inhibitor sorafenib was administered. Nevertheless, the patient died of cancer 12 months postoperatively.

### Case 2 (The husband of Case 1)

**Figure 3:**
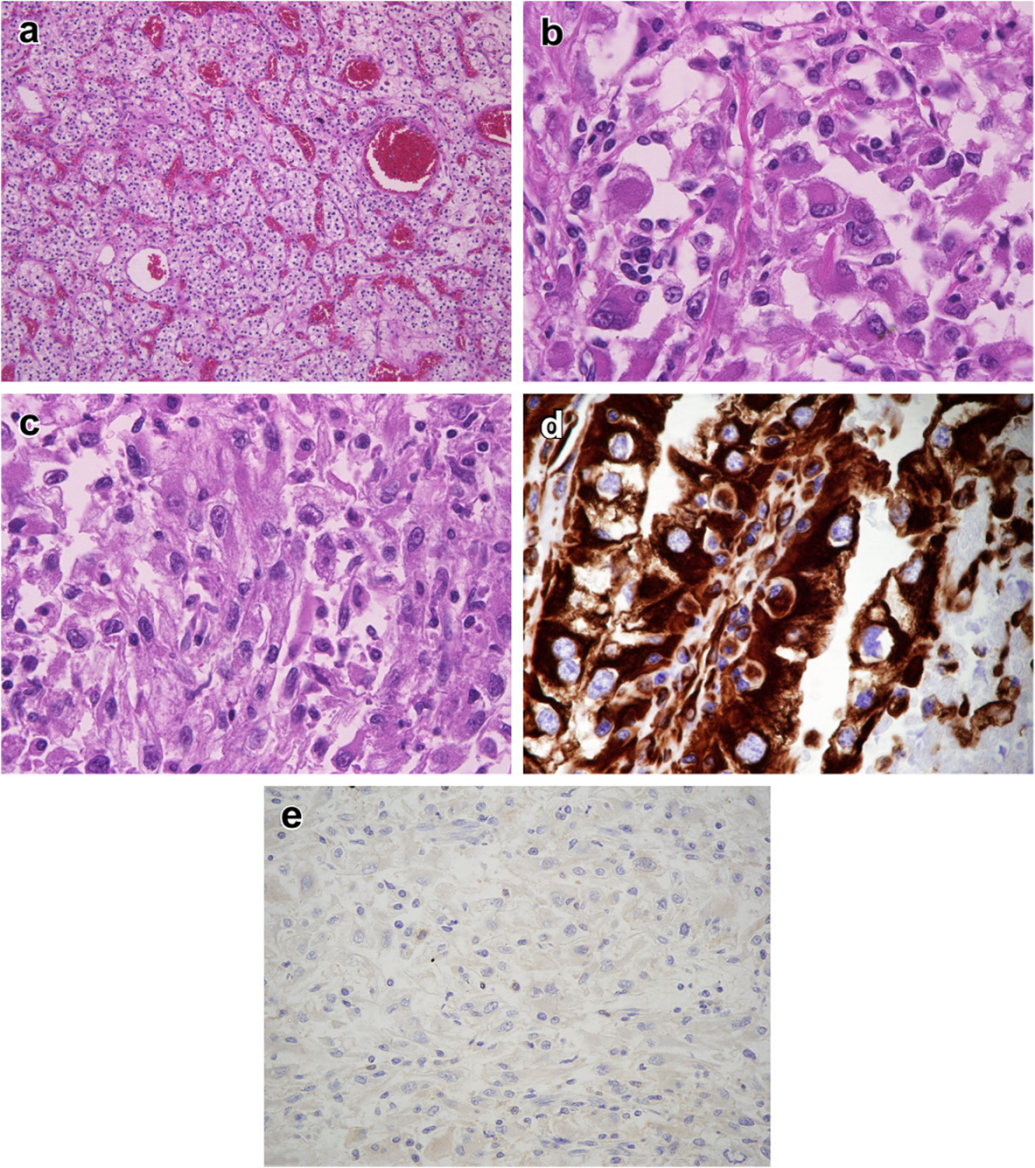
**Pathological findings in Case 2 (the husband of Case 1). (a)** Histological section displaying typical clear cell RCC area, **(b)** neoplastic cells with rhabdoid features, **(c)** spindle neoplastic cells, and **(d)** strong cytoplasmic positivity for vimentin. **(e)** BAP1 immunohistochemistry showed loss of nuclear staining in tumor cells.

In December 2009, the above patient’s 76-year-old husband with gross hematuria and was referred to our hospital. Enhanced CT scans of the abdomen revealed an 80 mm mass in his right kidney and tumor thrombus into the inferior vena cava (Figure 1b). In January 2010, right radical nephrectomy with vena caval thrombectomy was performed without any intraoperative or postoperative complications. Histological evaluation of the tumor denoted Fuhrman nuclear grade 4 clear cell RCC with rhabdoid features and sarcomatoid change (Figure 3a, b and c). Immunohistochemically, the rhabdoid cells were positive for vimentin, whereas the tumor cell nuclei were mostly negative for BAP1 (Figure 3d and e). In January 2011, the patient complained of lower back pain and neurological disorder of the lower extremity. Magnetic resonance imaging revealed vertebral metastasis at the L1 level and compression of the spinal cord. The patient died of an unrelated cause in April 2011.

### Background factors of cases 1 and 2

The man and women whose medical cases are described above have been married for >40 years. Neither of them had any remarkable medical history. Their daughter had died of pancreatic cancer, but parents, siblings, and their son had no history of malignancy. The husband was a shipyard worker and had been exposed to asbestos for >30 years. Additionally, the chest CT scan revealed that he had slight light pleural thickening. Although his wife was not employed, she routinely came into contact with his hair and clothing and could have been exposed to asbestos. The couple did not report any other unusual occupational exposures, such as organic solvents or cadmium. The couple had lived in Hokuto city for 50 years, and there was no report of significant increases in the incidence of RCC within that area.

## Conclusions

Familial RCC is well known, including von Hippel-Lindau disease and Birt-Hogg-Dube syndrome. However, spousal RCC has not been reported previously. The synchronous occurrence of RCC with rhabdoid features in this married couple is unlikely to have been purely coincidental. Exposure to asbestos has generally been associated with malignant mesothelioma in humans. It has long been recognized that some patients with mesothelioma result from household exposure to asbestos that is brought home in workers’ hair, clothing, and personal effects [5]. In a study of the wives of asbestos-exposed workers, Miller reported a mean latency of 45.4 years between the year of first exposure and the onset of symptoms of malignant mesothelioma [5]. RCC is also considered an occupationally associated tumor, but the existence of an association between RCC and asbestos exposure is currently controversial [6-10]. A recent genetics study of RCC found that rhabdoid features and high grade were associated with loss of BAP1, a nuclear deubiquitinase that regulates cell proliferation [11]. BAP1 copies are also lost in mesothelioma [12,13]. Furthermore, Carbone et al. have speculated that BAP1 may help prevent environmental carcinogenesis caused by asbestos [14]. Additionally, a few patients with concomitant RCC and malignant mesothelioma have been reported in Japan, although the reports of these cases have not indicated whether the RCC histologies were clear cell carcinoma with rhabdoid features [15]. In these cases, the development of RCC also occurred several decades after asbestos exposure. However, pathogenesis was not discussed in the associated case reports. Jiang et al. identified several novel characteristics of asbestos fibers in asbestos-induced oxidative DNA damage. By studying intraperitoneal administration of asbestos fibers in rats, Jiang et al. also found that the kidney was oxidatively stressed by asbestos fibers [16]. These results indicate that oxidative DNA damage by asbestos is a potential cause of RCC onset. In consideration of these findings, asbestos exposure and BAP1 loss appeared to result in very rare cases of spousal RCC.

Although other environmental exposures, including infection, cannot be completely ruled out and limited epidemiological evidence exists for the occurrence of asbestos-triggered RCC, asbestos exposure and BAP1 loss might have contributed to concurrent occurrence of RCC with rhabdoid features in this married couple. Future studies are necessary to elucidate the role of asbestos in the pathogenesis and progression of RCC with rhabdoid features.

## Consent

Written informed consent was obtained from a son of the patients (married couple) for publication of this Case report and any accompanying images. A copy of the written consent is available for review by the Editor of this journal.

## Abbreviations

RCC: Renal cell carcinoma
CT: Computed tomography

## References

[1] Gokden N, Nappi O, Swanson PE, Pfeifer JD, Vollmer RT, Wick MR (2000). Renal cell carcinoma with rhabdoid features. Am J Surg Pathol.

[2] Shannon B, Wisniewski ZS, Bentel J, Cohen RJ (2002). Adult rhabdoid renal cell carcinoma. Arch Pathol Lab Med.

[3] Kuroiwa K, Kinoshita Y, Shiratsuchi H, Oshiro Y, Tamiya S, Oda Y (2002). Renal cell carcinoma with rhabdoid features: an aggressive neoplasm. Histopathology.

[4] Leroy X, Zini L, Boub D, Ballereau C, Villers A, Aubert S (2007). Renal cell carcinoma with rhabdoid features. An aggressive neoplasm with overexpression of p53. Arch Pathol Lab Med.

[5] Miller A (2005). Mesothelioma in household members of asbestos-exposed workers: 32 United States cases since 1990. Am J Ind Med.

[6] Mandel JS, McLaughlin JK, Schlehofer B, Mellemgaard A, Helmert U, Lindblad P (1995). International renal-cell cancer study. IV. Occupation. Int J Cancer.

[7] Sali D, Boffetta P (2000). Kidney cancer and occupational exposure to asbestos: a meta-analysis of occupational cohort studies. Canc Causes Contr.

[8] Pesch B, Haerting J, Ranft U, Klimpel A, Oelschlägel B, Schill W (2000). Occupational risk factors for renal cell carcinoma:agent-specific results from a case–control study in Germany. MURC Study Group. Multicenter urothelial and renal cancer study. Int J Epidemiol.

[9] Mattioli S, Truffelli D, Baldasseroni A, Risi A, Marchesini B, Giacomini C (2002). Occupational risk factors for renal cell cancer: a case–control study in northern Italy. J Occup Environ Med.

[10] Karami S, Boffetta P, Stewart PS, Brennan P, Zaridze D, Matveev V (2011). Occupational exposure to dusts and risk of renal cell carcinoma. Br J Cancer.

[11] Peña-Llopis S, Vega-Rubín-de-Celis S, Liao A, Leng N, Pavía-Jiménez A, Wang S (2012). BAP1 loss defines a new class of renal cell carcinoma. Nat Genet.

[12] Bott M, Brevet M, Taylor BS, Shimizu S, Ito T, Wang L (2011). The nuclear deubiquitinase BAP1 is commonly inactivated by somatic mutations and 3p21.1 losses in malignant pleural mesothelioma. Nat Genet.

[13] Testa JR, Cheung M, Pei J, Below JE, Tan Y, Sementino E (2011). Germline BAP1 mutations predispose to malignant mesothelioma. Nat Genet.

[14] Carbone M, Yang H (2012). Molecular pathways: targeting mechanisms of asbestos and erionite carcinogenesis in mesothelioma. Clin Cancer Res.

[15] Sawazaki H, Yoshikawa T, Takahashi T, Taki Y, Takeuchi H, Sakai Y (2007). Renal cell carcinoma with malignant pleural mesothelioma after asbestos exposure: a case report. Hinyokika Kiyo.

[16] Jiang L, Nagai H, Ohara H, Hara S, Tachibana M, Hirano S (2008). Characteristics and modifying factors of asbestos-induced oxidative DNA damage. Cancer Sci.

